# Investigation of the new non-invasive semi-quantitative method of ^123^I-IMP pediatric cerebral perfusion SPECT

**DOI:** 10.1371/journal.pone.0241987

**Published:** 2020-11-09

**Authors:** Yasuharu Wakabayashi, Mayuki Uchiyama, Hiromitsu Daisaki, Makoto Matsumoto, Masafumi Sakamoto, Kenichi Kashikura

**Affiliations:** 1 Division of Radiological Technology, Saitama Prefectural Children's Medical Center, Saitama, Saitama, Japan; 2 Graduate School of Radiological Technology, Gunma Prefectural College of Health Science, Maebashi, Gunma, Japan; 3 Division of Radiology, Tokyo Jikeikai Medical University, Minato-ku, Tokyo, Japan; 4 Division of Radiological Technology, Saitama Prefectural Respiratory and Cardiovascular Center, Kumagaya, Saitama, Japan; Lee Kong Chian School of Medicine, SINGAPORE

## Abstract

In pediatric cases requiring quantification of cerebral blood flow (CBF) using ^123^I-N-isopropyl-p-iodoamphetamine (^123^I-IMP) single-photon emission computed tomography (SPECT), arterial blood sampling is sometimes impossible due to issues such as movement, crying, or body motion. If arterial blood sampling fails, quantitative diagnostic assessment becomes impossible despite radiation exposure. We devised a new easy non-invasive microsphere (e-NIMS) method using whole-body scan data. This method can be used in conjunction with autoradiography (ARG) and can provide supportive data for invasive CBF quantification. In this study, we examined the usefulness of e-NIMS for pediatric cerebral perfusion semi-quantitative SPECT and compared it with the invasive ARG. The e-NIMS estimates cardiac output (CO) using whole-body acquisition data after ^123^I-IMP injection and the body surface area from calculation formula. A whole-body scan was performed 5 minutes after the ^123^I-IMP injection and CO was estimated by region of interest (ROI) counts measured for the whole body, lungs, and brain using the whole-body anterior image. The mean CBF (mCBF) was compared with that acquired via ARG in 115 pediatric patients with suspected cerebrovascular disorders (age 0–15 years). Although the mCBF estimated by the e-NIMS indicated a slight deviation in the extremely low- or high-mCBF cases when compared with the values acquired using the invasive ARG, there was a good correlation between the two methods (r = 0.799; *p* < 0.001). There were no significant differences in the mCBF values based on physical features, such as patients’ height, weight, and age. Our findings suggest that ^123^I-IMP brain perfusion SPECT with e-NIMS is the simplest semi-quantitative method that can provide supportive data for invasive CBF quantification. This method may be useful, especially in pediatric brain perfusion SPECT, when blood sampling or identifying pulmonary arteries for CO estimation using the graph plot method is difficult.

## Introduction

Cerebral blood flow (CBF) distribution is widely evaluated via scintigraphy in various clinical situations such as in the diagnosis of acute encephalitis and encephalopathy, detection of the abnormal blood flow accompanying cerebrovascular disorders, circulation reserve evaluation by the Acetazolamide stress test, and for understanding the neuropsychiatric disorder represented by epilepsy. The neutral lipophilic substance, ^123^I-N-isopropyl-p-iodoamphetamine (^123^I-IMP), has pharmacokinetic properties that are more quantitative than other radiopharmaceuticals for CBF evaluation using single-photon emission computed tomography (SPECT). After an intravenous injection, ^123^I-IMP temporarily accumulates in the lungs before being rapidly released into the arteries. Moreover, it accumulates at a high rate, 90% or more, in the brain tissue in the first circulation and is distributed proportionally to the regional CBF (rCBF). The peak time of accumulation occurs 15–30 minutes after an intravenous injection; afterwards, it is slowly washed out of the brain [1–3]. Since early washout after injection can be ignored, the microsphere model was established. The microsphere method can obtain rCBF by continuous arterial blood sampling to obtain the integral value of the input function and dividing the SPECT count in the brain by the integral value of the input function. The input function of the microsphere method requires actual measurement of continuous arterial blood sampling, whereas the NIMS method measures the injection dose, lung washout rate, and cardiac output based on planar imaging counts and uses them to estimate the input function without arterial blood sampling.

Although invasive CBF evaluated by ^123^I-IMP SPECT is generally quantified using arterial blood sampling methods, it is sometimes difficult to perform pediatric brain perfusion SPECT with an arterial puncture because of crying, body movements, or the patient waking up from sedation. Therefore, continuous blood sampling methods present a puncture injury risk [4, 5]. The one-point arterial blood sampling method, also known as the autoradiography method (ARG), is the most recommended and conventional approach for quantitative pediatric brain perfusion SPECT [6, 7].

However, even with ARG, there are cases in which blood sampling is abandoned when severe body movements result in an inability to find the arteries. Furthermore, the quantitative accuracy of ARG may decline due to venous blood contamination or deviation from the recommended sampling time. In pediatric patients in whom blood sampling becomes impossible, attempts to achieve CBF quantification are abandoned. As a result, it becomes impossible to perform a quantitative diagnostic assessment despite radiation exposure.

Currently, non-invasive, semi-quantitative methods are not widely used because their quantitative accuracy is inferior to blood sampling methods. However, invasive methods often fail even in adults. In contrast, non-invasive methods are not affected by various error factors caused by blood sampling and can be performed easily. For example, in the non-invasive microsphere (NIMS) method, the cardiac output (CO) index is calculated from the counts within a region of interest (ROI) located in both the right ventricle and pulmonary artery [8]. Although it is possible to estimate the CO by considering the washout from the lungs and obtain the integral value of the input function to the brain with this method, dynamic planar acquisitions are required in addition to brain SPECT acquisitions. When NIMS is successful, the rCBF can be calculated as follows:

$$rCBF=\frac{CCF\times Cb\left(SPECT\right)\times \frac{B(5)}{B(20)}\times CO\times 100}{Q\times (1-\frac{L(5)}{Lmax})\times 1.04}\;(mL/100\;g/min)$$ (1)

where CCF is the cross-calibration factor, Cb is the count per voxel acquired via brain SPECT, B(5) is the planar count of the brain 5 minutes after injection, B(20) is the planar count of the brain 20 minutes after injection, Q is the ^123^I-IMP all-count value of the syringe before injection, L(5) is the planar count of the lungs 5 minutes after injection, Lmax is the maximum count of the lungs up to 5 minutes after injection, and 1.04 (g/mL) is the density of the brain tissue [9].

In the fractional uptake (FU) method, the mean CBF (mCBF) of the whole brain is measured by the product of the CO flow and FU [10], as follows:

mCBF=CO×FU (2)

In order to calculate the CO flow non-invasively, both the FU and NIMS methods require a constant ^123^I-IMP injection rate. This procedure extends the scan time required for examination and complicates the overall process. Pediatric patients typically receive fewer injected doses and show higher heart rates than adults [11]. In addition, setting the ROIs for a CO flow calculation is extremely difficult due to the small pulmonary arteries and small left ventricle of pediatric patients [12]. It is not practical to extend both examination and sedation times for pediatric patients by adding multiple SPECT and planar acquisitions that are needed for non-invasive methods. For these reasons, we developed an easy non-invasive microsphere (e-NIMS) method as a new non-invasive semi-quantitative technique using whole-body scans. The e-NIMS simply estimates the CO using both the body surface area (BSA) and planar counts calculated by the whole-body scan immediately after the ^123^I-IMP injection. A constant ^123^I-IMP injection rate is not required like the FU and NIMS methods. The e-NIMS method is a simpler procedure than those methods. In this study, we compared our proposed e-NIMS with the conventional invasive ARG in order to examine its validity for the simple estimation of pediatric CBF in SPECT evaluations.

## Materials and method

### Patient characteristics

The study protocol was reviewed and approved by the institutional ethics review boards of Saitama children's medical center (approval number 2017-99-002). All patients or parents provided comprehensive informed consent for a series of examinations and analysis. We investigated a total of 115 children (age: 0–15 years, 65 boys and 50 girls) who had undergone ^123^I-IMP (FUJIFILM Toyama Kagaku Co. Ltd, Tokyo, Japan) CBF SPECT between April 2016 and December 2017 (Table 1). The height and weight range of these patients were 50.0–165.3 cm and 3.3–65.2 kg, respectively. Injected doses of ^123^I-IMP ranged from 58.3 to 187.9 MBq [13]. As per the Japanese consensus guidelines for pediatric nuclear medicine, potassium iodide was administered for 4 days to avoid thyroid uptake 2 days before ^123^I-IMP CBF SPECT [14].

**Table 1:** Clinical diagnosis of subjects.

Diagnostic category	Number of cases
Epilepsy	58
West syndrome	18
Dravet syndrome	3
BECT	2
Acute encephalitis	7
Limbic encephalitis	5
SLE	2
Other	20
Total	115

BECT: benign childhood epilepsy, SLE: systemic lupus erythematosus.

### Equipment and SPECT imaging protocol

The Symbia E SPECT scanner (Siemens, Illinois, USA) fitted with a low-medium energy general-purpose collimator was used in this study. Syngo MI VA 10 D (Siemens, Illinois, USA) and a three-dimensional stereotaxic ROI template (3DSRT neuro, FUJIFILM Toyama Kagaku Co. Ltd, Tokyo, Japan) were used for data analysis [15]. ATOMLAB 100 Plus (BIODEX, New York, USA) was used as a dose calibrator, and TDC-521 (Hitachi, Tokyo, Japan) was used as a gamma-ray well-scintillation counter. Calibration and sufficient quality assurance measures were performed for all measuring devices before the study.

The CCF between the Symbia E SPECT scanner and well-scintillation counter was determined under the same conditions as for clinical acquisitions, using a 15-cm long cylinder phantom (Molecular Imaging Labo Inc., Osaka, Japan) with a 16-cm inner diameter. SPECT acquisition was performed with a mid-scan time of 30 minutes after the ^123^I-IMP injection [7] and was conducted for 24 minutes (1 cycle per 3 minutes, 8 repeats) using a 180-degree continuous mode in a circular orbit with a 14-cm radius.

**Fig 1:**
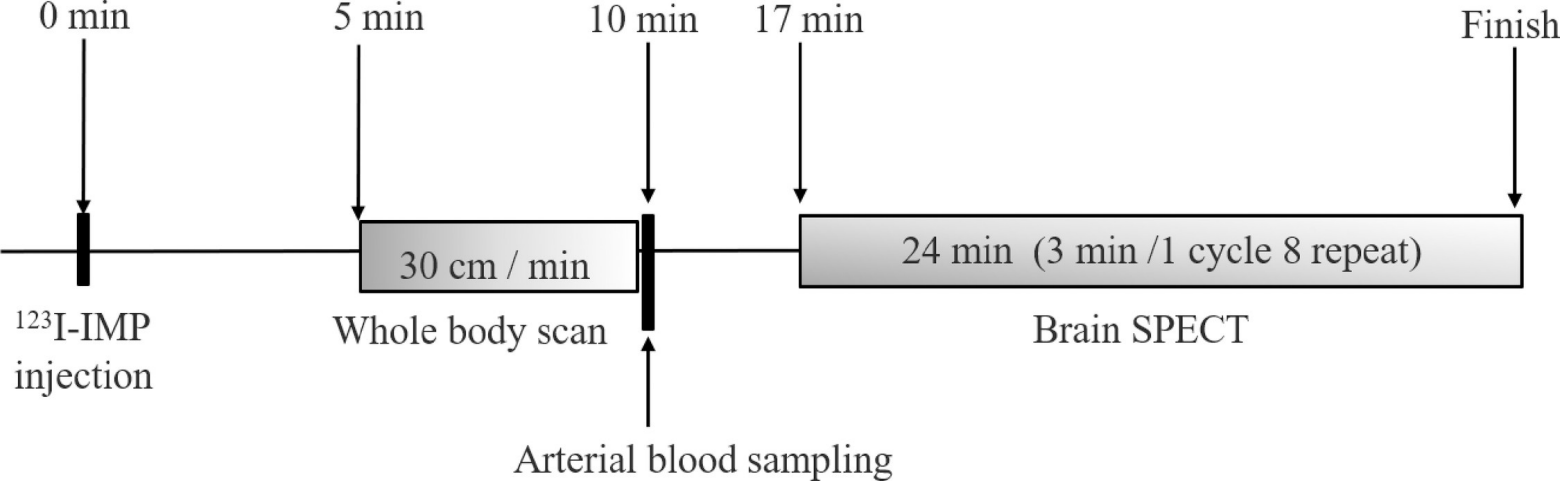
Timeline of the protocols, including the whole-body fractional uptake and autoradiography.

The matrix size was 128 × 128, and the zoom factor was set at 2.0; as a result, the pixel size was 2.4 mm. The energy window was set at 159 keV ± 15%. All image reconstructions were performed using a filtered back projection algorithm, and attenuation corrections were applied using Chang’s method with a 0.08-cm^-1^ attenuation coefficient; scatter correction was not applied. The Butterworth filter with an order of 8 and a cut-off value of 0.25 cycles/cm was applied prior to performing image reconstructions to reduce image noise. The imaging protocol of this study is shown in Fig 1.

### ARG

The ARG, which is based on 1-point arterial blood sampling, was evaluated as an invasive method. This method assumes a 2-compartment model, and the mCBF is estimated by considering the washout from the brain. Arterial blood (1 mL) was sampled 10 minutes after the ^123^I-IMP injection, and gamma-ray counts of the sample were measured for 5 minutes using a gamma-ray well scintillation counter. The standard input function data of Akita Research Institute of Brain and Blood Vessels [2] was preloaded on Syngo MI VA 10 D. In this study, the distribution volume (Vd) of ^123^I-IMP was assumed to be 45 mL/mL [7, 16, 17].

### e-NIMS

**Fig 2:**
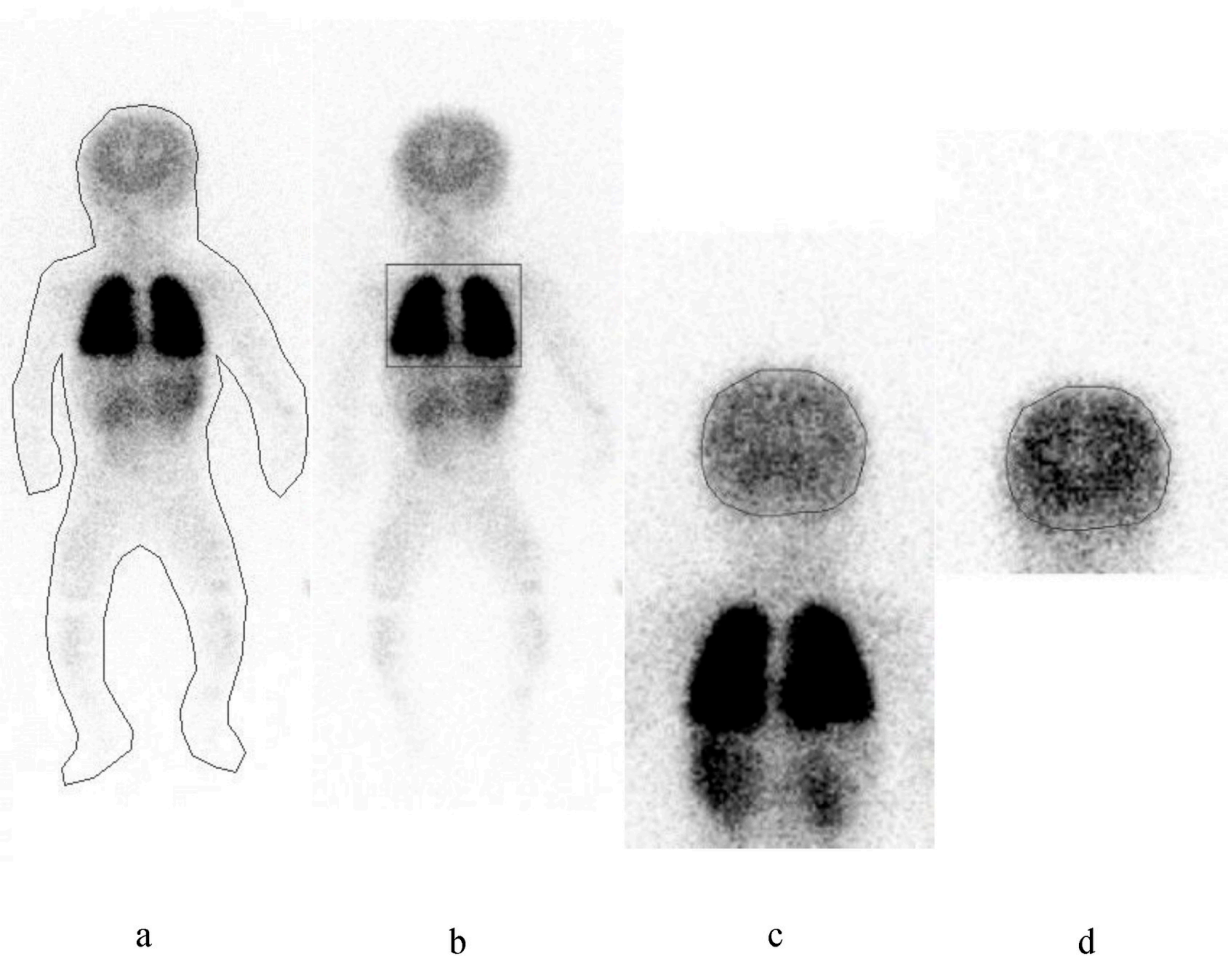
Representative image for setting the region of interest with accumulation count for each ROI setting. **a)** ROI count for the whole-body (WB). **b)** ROI count for the lung in the WB scan image. **c**) ROI count for the brain in the WB scan image. **d)** ROI count for the brain in single-photon emission computed tomography image. ROI, region of interest.

A whole-body scan was performed 5 minutes after injection of ^123^I-IMP. Data were acquired from the foot to the head using a scan speed of 30 cm/min, and the ROI counts were measured for the whole-body, lungs, and brain, using the whole-body anterior image (Fig 2).

In the e-NIMS, the following CCF(e-NIMS) value was substituted into the CCF in Eq (1) to estimate the mCBF:

$$CCF(eNIMS)=\frac{phantom\;count\;with\;whole-body\;scan\;[count]}{phantom\;volume[ml]\times mean\;SPECT\;phantom\;count[count/ml]}$$ (3)

where the phantom count with the whole-body scan is the count per voxel of cylinder phantom acquired via whole-body scan, similarly to the acquisition protocol, and the mean SPECT phantom count is the count per voxel of cylinder phantom acquired via SPECT scan. The phantom volume is calculated in ml.

Substitute the following into Eq (1) and calculate. B (5) is substituted for the brain ROI counts of the whole-body scan 5 minutes after the injection, multiplied by the CCF, and B (20) is substituted for the brain ROI counts in the SPECT projection.

CO in Eq (2) is calculated from the BSA and cardiac index (CI) as follows:

$$CO[ml/min]=CI\;[l/min{/m}^{2}]\times BSA[m^{2}]\times 100$$ (4)

where CI is set to its standard value of 3.5. Q in Eq (1) is substituted for the planar counts of the whole-body scan 5 minutes after the injection. L (5) in Eq (1) is substituted for the lung ROI count from the whole-body scan 5 minutes after the injection, and Lmax in Eq (1) is substituted for the counts of the whole-body planar image 5 minutes after the injection. The BSA in Eq (4) was calculated using Fujimoto’s formula [18] in all cases, as follows:

$$BSA\;[m^{2}]={weight\;[kg]}^{0.444}\times {height\;[cm]}^{0.663}\times 0.008883.$$ (5)

### Data analysis

All data are presented as the mean ± standard deviation. Pearson correlation analysis was used to evaluate the relationship between mCBF values calculated by the e-NIMS and the ARG. The paired t-test was used to evaluate significant differences. *P* values < 0.05 were considered statistically significant. The analysis of descriptive statistics and basic comparisons were carried out using the EZR (Easy R version 1.38, Saitama Medical Center, Jichi Medical University, Saitama, Japan) software.

## Results

**Fig 3:**
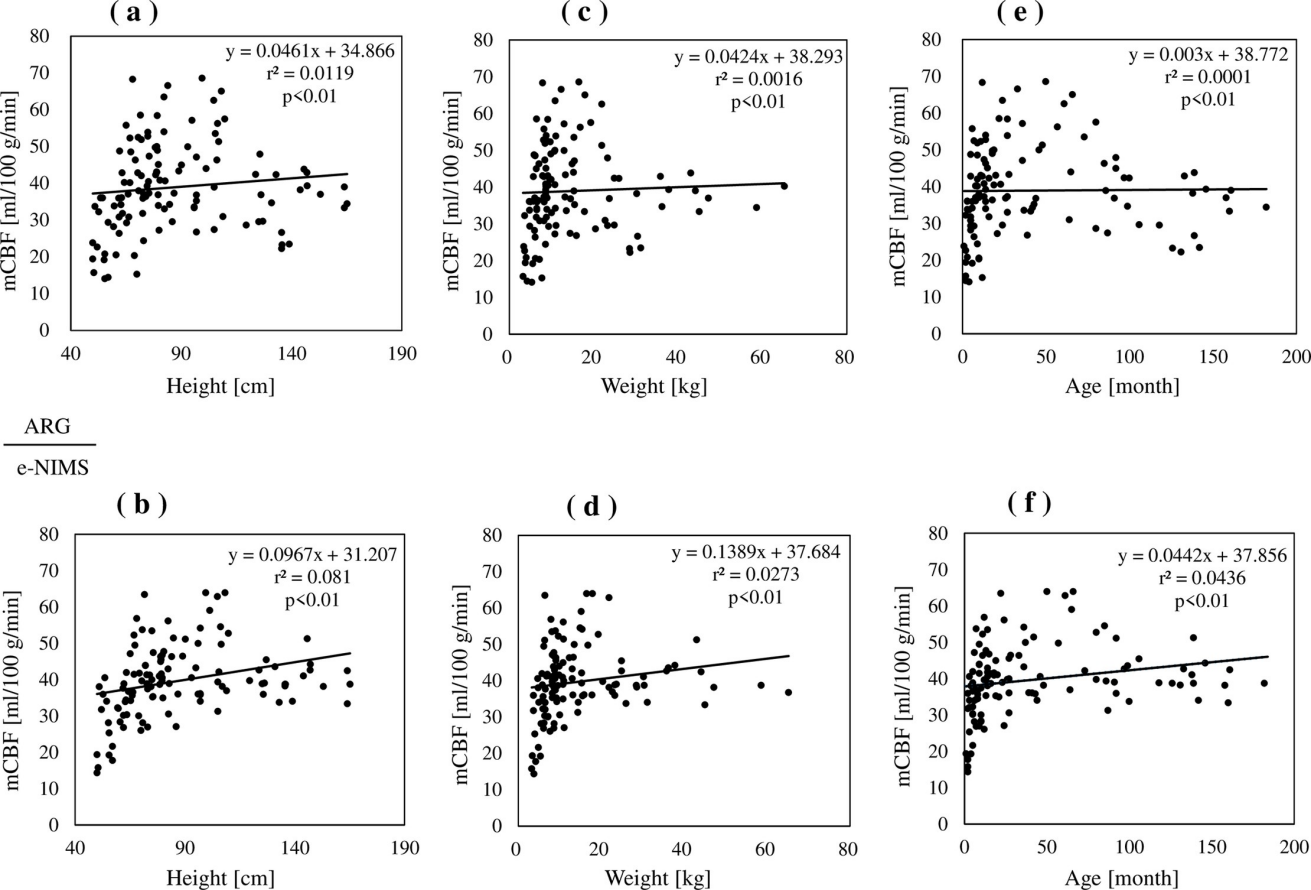
Mean cerebral blood flow values obtained using whole-body fractional uptake and autoradiography. **a, b)** mCBF distribution by height. **c, d)** mCBF distribution by weight. **e, f)** mCBF distribution by age.

**Fig 4:**
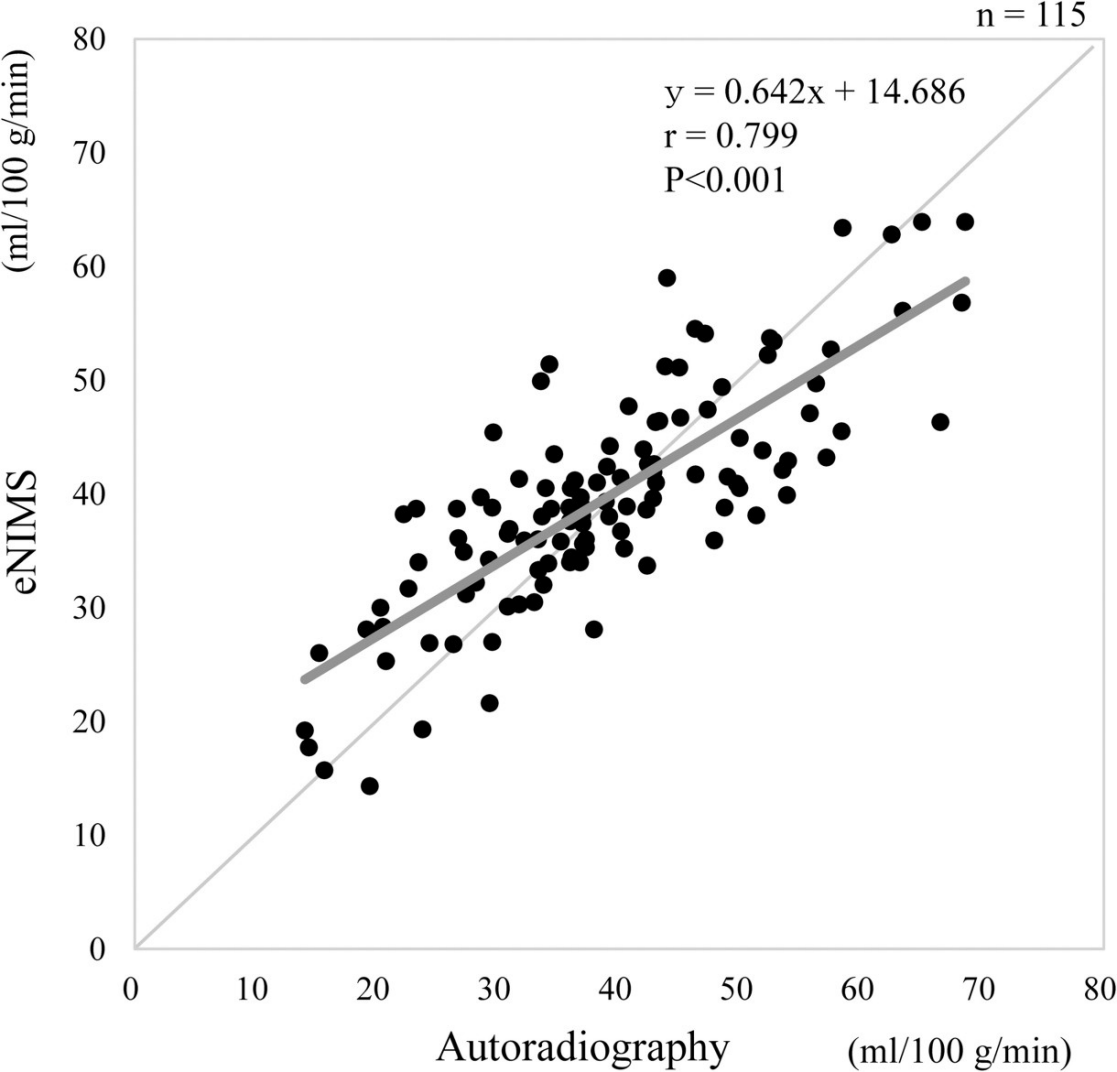
Comparison of mean cerebral blood flow values obtained by easy-noninvasive micro sphere and autoradiography.

**Fig 5:**
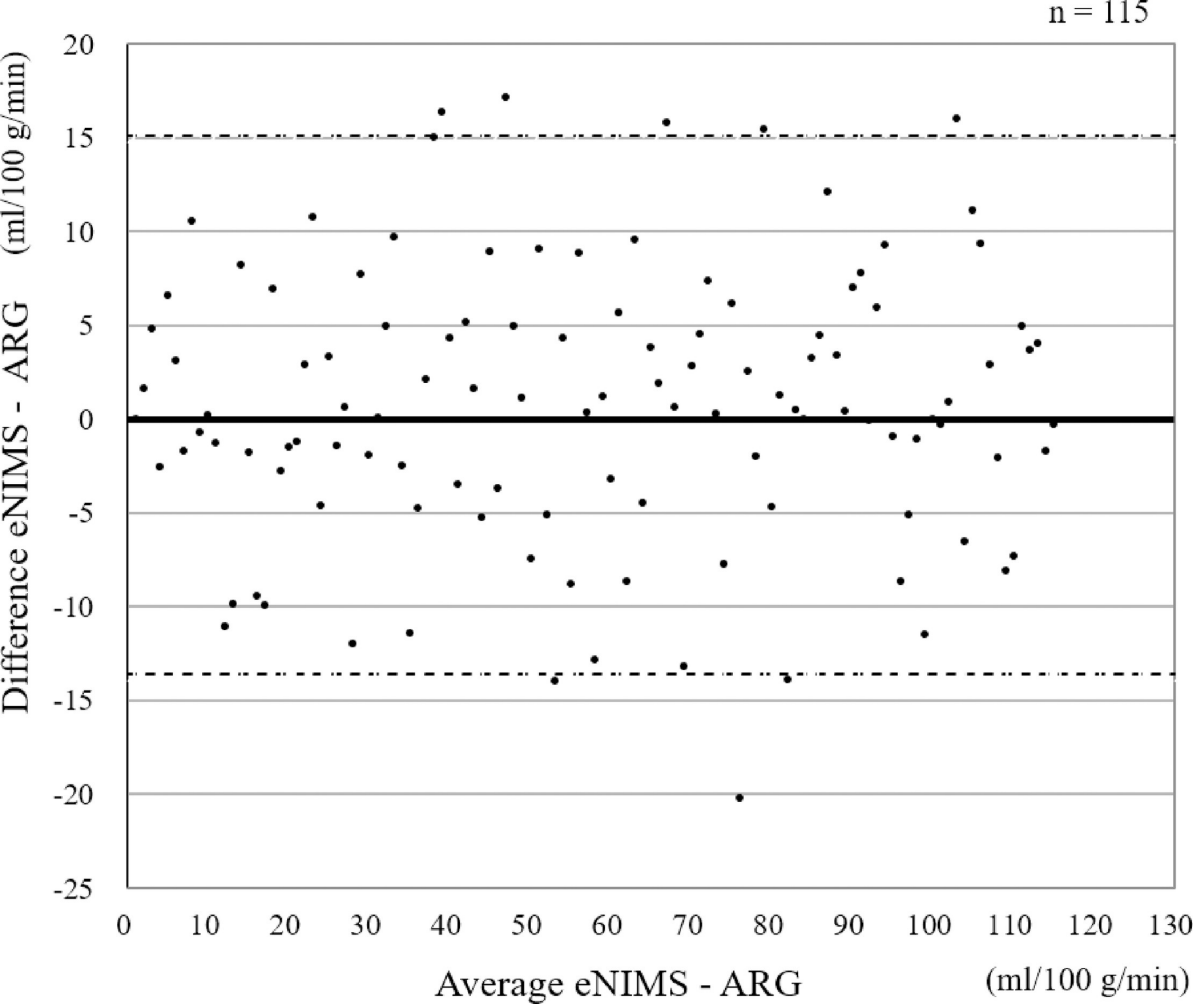
Bland-Altman plot for comparison of easy non-invasive micro sphere and autoradiography. Small dashed line denotes the limits of agreement (± 1.96 SD).

The comparison of mCBF values measured by the e-NIMS and ARG regarding several physical factors, such as height, weight, and age, is shown in Fig 3. There were no significant correlations between the mCBF and physical factors in any of the indices measured. The mCBF was 38.9 ± 12.2 mL/100 g/min for the ARG and 39.7 ± 9.8 mL/100 g/min for e-NIMS; there were no significant differences in mCBF values between the two methods. Fig 4 shows the comparison of mCBF values obtained by e-NIMS and ARG, indicating a correlation between the two methods (correlation coefficient: *r* = 0.799, *p* < 0.001). In cases with low blood flow, the mCBF tended to be slightly higher when measured by the e-NIMS than when measured by the ARG. In contrast, when the blood flow measured by ARG was high, the mCBF tended to be lower with the e-NIMS than with the ARG. In several cases where the ARG was used, mCBF deviations may have occurred because of the failure to collect blood samples. Bland-Altman plots of mCBF values obtained via e-NIMS and ARG methods was shown in Fig 5. The figure shows a scatter diagram of the differences plotted against the averages of the two measurements. Horizontal lines are drawn at the mean difference and at the limits of agreement defined as the mean difference plus and minus 1.96 times the standard deviation of the differences. The mean difference was 0.76 and limit of agreement ranged from -13.57 to 15.09 ml/100 g/min. The relationship between e-NIMS and ARG is considered good.

## Discussion

It is often risk and extremely difficult to perform invasive brain perfusion SPECT in children than in adults; therefore, we considered that non-invasive methods which substitute conventional quantitative methods would be preferable for children. As demonstrated in this study, e-NIMS utilizes whole-body distribution counts after ^123^I-IMP injection and is a simple, semi-quantitative evaluation method, which requires the patients to undergo only one whole-body imaging scan immediately before performing the ARG method. One possible reason is the difficulty to assure quantitative accuracy because of numerous variables that need to be considered for pediatric patients. For example, the volume and perfusion of the brain changes drastically between neonatal and infant periods due to growth [19]. It is also difficult to generate normal databases since data on healthy children are rare. Therefore, even if pediatric CBF values are obtained using a strict quantitative method, it remains unclear whether the values fall within normal or abnormal ranges. On the other hand, various invasive quantification methods have shown accurate mCBF values in adults. However, these invasive methods cannot provide accurate quantitative estimation when arterial blood sampling cannot be performed at the preferred time, and especially in children in whom arterial blood sampling is extremely difficult.

The most frequently occurring errors with ARG in this study are as follows: (1) incomplete CCF setting, (2) deviations in the blood sampling time after injection, and (3) failure to obtain arterial blood. In this study, the appropriate CCF was determined before the study was conducted. The time of arterial blood sampling deviated from the target time of 10 minutes after the injection in several cases, ranging between −1 and +26 minutes. This deviation is also indicative of a shift of mid-scan time. Moreover, accidental puncture of a vein close to the artery due to inadvertent body movements may contaminate the required blood sample with venous blood. Therefore, a non-invasive method using ^123^I-IMP SPECT that does not require arterial blood sampling is considered ideal for CBF evaluation in pediatric patients with difficult characteristics.

The graph plot method that sets the ROI on the pulmonary artery to obtain an input function was recently developed for use as a non-invasive method [20–22]. However, it is not ideal to apply the graph plot method for pediatric patients which it may be difficult to identify the small pulmonary artery because of the low counts with regard to high heart rate and low dose in children. In contrast, e-NIMS allows the objective evaluation of CBF and is not affected by low dosage, high heart rate, or manual error of ROI setting for CO calculation.

Several previous studies have used ARG to compare other potentially advantageous methods. Kaminaga et al. reported a significant correlation between the non-invasive NIMS method and ARG [23]. Abe et al. reported a significant correlation between the mCBF obtained by ARG and that acquired using ^15^O-H_2_O positron emission tomography [24]. Based on the results of these studies, we also considered it prudent to compare the results of e-NIMS and ARG. Our results indicated good correlation between semi-quantitative e-NIMS and quantitative ARG for evaluating pediatric mCBF. However, ARG does not provide an absolute quantification value.

This study potentially has some errors and limitations with the e-NIMS and ARG [16]. Both the standardized arterial input function and the Vd in ARG have been devised for adults [7, 16]. No previous study has reported the validity of mCBF values in children using the coefficient values obtained from adults. However, ARG is often used in pediatric CBF SPECT because the calculated CBF is clinically used as the reference value for each pediatric patient, even if the quantitative evaluation is performed using ARG. Further, the limitations of semi-quantitative e-NIMS method include the physical factors (such as age and height), differences in internal circulation because of age and disease, and the accuracy of BSA calculation for CO estimation. Notably, the start and end times of the whole-body scan are different among different individuals because they are affected by each patient’s characteristics, such as height, sedation status, and level of movement. The ROI counts of the lungs also differ for each individual because lung accumulation changes with internal circulation [25]. Deviations in the counts evaluated in whole-body scans due to these factors can lead to quantitative errors. There are various BSA calculation formulas, such as the Dubois or Haycock’s formula [26, 27]. Herein, we used the Fujimoto formula, which is the most frequently used formula in Japanese pediatric hospitals. Although the mCBF with e-NIMS obtained using the Fujimoto formula showed good correlation with ARG, we consider that additional studies involving the calculation of CO values using other BSA calculation formulas are necessary. Since our study data included pediatric patients with illness and not healthy adults, a more detailed evaluation using a varied patient population is warranted. The resolution of SPECT or whole-body scan can influence CBF evaluation. Although resolution correction was not applied in this study, it is necessary to examine the effect of SPECT scanners with resolution correction on CBF evaluation in the future. The recent widespread use of hybrid SPECT allows the use of CT or MRI-based attenuation and scatter correction [28, 29], which could further improve CBF quantification of various non-invasive methods, including e-NIMS. Recently, MRI using arterial spin-labeling (ASL) has been used for CBF evaluation [30–32]. CBF measurement by ASL has the advantages of no radiation exposure and minimal invasiveness in pediatric patients. However, it may be desirable to use standard evaluation methods together, such as cerebral perfusion SPECT and ^15^O-PET, owing to metal artifacts and difficulty in the cerebellar blood flow estimation [33], etc. Although the evaluation of pediatric CBF using ASL may be expanded in the future, comparison with a conventional method of CBF-SPECT with sufficient samples will be necessary.

## Conclusions

We devised the e-NIMS as a new, non-invasive, semi-quantitative approach for pediatric brain perfusion SPECT using ^123^I-IMP and compared its results with those of the invasive ARG. After examining 115 pediatric patients, the e-NIMS tended to slightly overestimate mCBF values in cases of very low CBF compared with the invasive ARG. In contrast, e-NIMS slightly underestimated mCBF values in cases of very high CBF compared to values acquired via ARG. However, the two methods were highly correlated (r = 0.799). The e-NIMS method is highly feasible and can be used in conjunction with the ARG; it can provide support for invasive CBF quantification failure. Particularly, we propose that the e-NIMS is useful for assessing mCBF in cases requiring pediatric brain perfusion SPECT, in which blood sampling and identifying pulmonary arteries for CO estimation using non-invasive methods such as the graph plot method, are difficult.

## References

[1] Kuhl DE, Barrio JR, Huang SC, Selin C, Ackermann RF, Lear JL, et al Quantifying local cerebral blood flow by N-isopropyl-P-[^123^I] iodoamphetamine (IMP) tomography. J Nucl Med. 1982;23:196–203. 6801219

[2] Iida H, Itoh H, Nakazawa M, Hatazawa J, Nishimura H, Onishi Y, et al Quantitative mapping of regional cerebral blood flow using iodine-123-IMP and SPECT. J Nucl Med. 1994;35:2019–30. 7989987

[3] Kim KM, Watabe H, Hayashi T, Hayashida K, Katafuchi T, Enomoto N, et al Quantitative mapping of basal and vasareactive cerebral blood flow using spilt-dose ^123^I-iodoamphetamine and single photon emission computed tomography. Neuroimage. 2006;33:1126–35. 10.1016/j.neuroimage.2006.06.064 17035048

[4] Iida H, Nakagawahara J, Hayashida K, Fukushima K, Watabe H, Koshino K, et al Multicenter evaluation of a standardized protocol for rest and acetazolamide CBF assessment using a quantitative SPECT reconstruction program and split-dose ^123^I-Iodoamphetamine. J Nucl Med. 2010;51:1624–31. 10.2967/jnumed.110.078352 20847163

[5] Matsuda H, Seki H, Ishida H, Sumiya H, Tsuji S, Hisada K, et al Regional cerebral blood flow measurement using N-isopropyl-p-[^123^I] iodoamphetamine and rotating gamma camera emission computed tomography. Kaku Igaku. 1985;22(1):9–18. 3873559

[6] Itou H, Iida H, Murakami M, Bloomfield PM, Miura S, Okudera T, et al A method for measurement of regional cerebral blood flow using N-isopropyl-p-[^123^I] iodoamphetamine (^123^I-IMP) SPECT; two scans with one point blood sampling technique. Kaku Igaku. 1992;29(10):1193–200. 1464958

[7] Iida H, Itoh H, Bloomfield PM, Munaka M, Higono S, Murakami M, et al A method to quantitate cerebral blood flow using a rotating gamma camera and iodine-123 iodoamphetamine with one blood sampling. Eur J Nucl Med. 1994;21(10):1072–84. 10.1007/BF00181062 7828617

[8] Yonekura Y, Sugihara H, Taniguchi Y, Aoki E, Furuichi K, Miyazaki Y. Quantification of brain perfusion SPECT with N-isopropyl-p-iodoamphetamine using non-invasive microsphere method: estimation of arterial input by dynamic imaging. Kaku Igaku. 1997;34(10):901–8. 9404097

[9] Rempp KA, Brix G, Wenz F, et al: Quantification of regional cerebral blood flow and volume with dynamic susceptibility contrast-enhanced MR imaging. Radiology 193: 637–641, 1994 10.1148/radiology.193.3.7972800 7972800

[10] Yonekura Y, Iwasaki Y, Fujita T, Sasayama T, Matoba N, Sadafuchi N, et al Simple quantification of brain perfusion SPECT with N-isopropyl-p-[^123^I] iodoamphetamine using a large field gamma camera. Kaku Igaku. 1990;27(11):1311–6. 2290200

[11] Massin M, von Bernuth G. Normal ranges of heart rate variability during infancy and childhood. Pediatr Cardiol. 1997;18(4):297–302. 10.1007/s002469900178 9175528

[12] Miyazaki Y, Hashimoto M, Kinuya S, Satake R, Inoue H, Shiozaki J, et al Modifications of fractional uptake method for ^123^I-IMP. Kaku Igaku. 1996; 33:285–91. 8622262

[13] **)** Subcommittee for Standardization of Radionuclide Imaging, Medical and Pharmaceutical Committee: Japan Radioisotope Association. Recommendation for pediatric dose in nuclear imaging. Radioisotopes. 1988; 37:627–32. 3222473

[14] Koizumi K, Masaki H, Matsuda H, Uchiyama M, Okuno M, Oguma E, et al Guideline for pediatric nuclear medicine. Ann Nucl Med. 2014; 28:498–503. 10.1007/s12149-014-0826-9 24647992 PMC4061477

[15] Takeuchi Ryo, Yonekura Yoshiharu, Matsuda Hiroshi, et al Usefulness of three-dimensional stereotaxic ROI template on anatomically standardized ^99m^Tc-ECD SPET. Eur J Nucl Med.2002; 29:331–341. 10.1007/s00259-001-0715-z 12002707

[16] Iida H, Nakazawa M, Umemura K. Quantitation of regional cerebral blood flow using ^123^I-IMP from a single SPECT scan and a single blood sampling: analysis on statistical error source and optimal scan time. Kaku Igaku. 1995;32(3):263–70. 7739156

[17] Nagamachi Shigeki, Jinnouchi Seishi, Nishii Ryuichi, Fujita Seigo, Futami Shigemi, Tamura Shozo, et al The Development of New Method for Assessment of Perfusion Reserve Using Split Dose Iodine-123-IMP SPECT: One-Day Protocol by Modified ARG Method.Kakuigaku.2003;40:155–162. 12884782

[18] Fujimoto S, Watanabe T, Yukawa K, Sakamoto J. Studies on the physical surface area of Japanese part 17 regional rates according to sex, age and body shape. Nihon Eiseigaku Zasshi. 1968;23(5):437–42. 10.1265/jjh.23.437 5752711

[19] Chiron C, Raynaud C, Mazière B, Zilbovicius M, Laflamme L, Masure MC, et al Changes in regional cerebral blood flow during brain maturation in children and adolescents. J Nucl Med. 1992; 33:696–703. 1569478

[20] Okamoto K, Ushijima Y, Okuyama C, Nakamura T, Nishimura T. Measurement of cerebral blood flow using graph plot analysis and I-123 iodoamphetamine. Clin Nucl Med. 2002;27:191–6. 10.1097/00003072-200203000-00009 11852307

[21] Ofuji A, Mimura H, Yamashita K, Takaki A, Sone T, Ito S. Development of a simple non-invasive microsphere quantification method for cerebral blood flow using I-123-IMP. Ann Nucl Med. 2016;30:242–9. 10.1007/s12149-015-1053-8 26733060

[22] Kameyama M, Watanabe K. A new non-invasive graphical method for quantification of cerebral blood flow with I-123-IMP. Ann Nucl Med. 2018;32:620–6. 10.1007/s12149-018-1282-8 30046997 PMC6208854

[23] Kaminaga T, Kunimatsu N, Chikamatsu T, Furui S. Validation of CBF measurement with non-invasive microsphere method (NIMS) compared with autoradiography method (ARG). Ann Nucl Med. 2001;15:61–4. 10.1007/BF03012134 11355785

[24] Abe S, Kato K, Takahashi Y, Fujita N, Yamashita M, Shinoda M, et al Estimation of ^123^I-IMP arterial blood activity using ^123^I-IMP acquisition data from the lungs and brain without any blood sampling. Clin Nucl Med. 2012;37(3):258–63. 10.1097/RLU.0b013e31823928a7 22310252

[25] Suga K, Ariyoshi I, Nakanishi T, Utsumi H, Yamada N. Clinical and experimental studies on the mechanism of abnormal accumulation in lung scanning with ^123^I-IMP. Nucl Med Commun. 1992;13(1):33–40. 10.1097/00006231-199201000-00006 1594167

[26] DuBois D, DuBois EF. A formula to estimate the approximate surface area if height and weight be known. Arch Intern Med. 1916;17:861–71.

[27] Haycock GB, Schwartz GJ, Wisotsky DH. Geometric method for measuring body surface area: A height-weight formula validated in infants, children and adults. J Pediatr. 1978; 93:62–66. 10.1016/s0022-3476(78)80601-5 650346

[28] Knoll Peter, Kotalova Daniela, Kochle Gunnar, Kuzelka Ivan, Minear Greg, et al Comparison of advanced iterative reconstruction methods for SPECT/CT. Medizinische Physik. 2012; 22(1): 58–69. 10.1016/j.zemedi.2011.04.007 21723716

[29] Wintermark Max, Sesay Musa, Barbier Emmanuel, Borbely Katalin, Dillon William P., Eastwood James D., et al Comparative Overview of Brain Perfusion Imaging Techniques. Stroke. 2005; 36: e83–e99. 16100027 10.1161/01.STR.0000177884.72657.8b

[30] David C. Alsop, John A. Detre. Multisection Cerebral Blood Flow MR Imaging with Continuous Arterial Spin Labeling. Radiology.1998; 208:410–416. 10.1148/radiology.208.2.9680569 9680569

[31] Biagi Laura, Abbruzzese Arturo, Maria Cristina Bianchi David C. Alsop, Alberto Del Guerra, et al Age Dependence of Cerebral Perfusion Assessed by Magnetic Resonance Continuous Arterial Spin Labeling. J Magn Reson Imaging.2007;25:696–702. 17279531 10.1002/jmri.20839

[32] Liu Feng, Duan Yunsuo, Peterson Bradley S., et al Resting state cerebral blood flow with arterial spin labeling MRI in developing human brains. Euro J Ped Neuro. 2018; 4:582–583. 10.1016/j.ejpn.2018.03.003 29656926

[33] Deibler A.R., Pollock J.M., Kraft R.A., Tan H., Burdette J.H. and Maldjian J.A. Arterial Spin-Labeling on Routine Clinical Practice, Part 1: Technique and Artifacts. AJNR. 2008; 29(7): 1228–1234. 10.3174/ajnr.A1030 18372417 PMC4686140

