# Correction: Investigation of the new non-invasive semi-quantitative method of 123I-IMP pediatric cerebral perfusion SPECT

**DOI:** 10.1371/journal.pone.0301961

**Published:** 2024-04-04

**Authors:** Yasuharu Wakabayashi, Mayuki Uchiyama, Hiromitsu Daisaki, Makoto Matsumoto, Masafumi Sakamoto, Kenichi Kashikura

In the e-NIMS subsection of the Materials and Method, there is an error in the second equation. The formula is incorrect. Please view the complete, correct equation here:

CO[ml/min]=CI[l/min/m2]×BSA[m2]×1000

Fig 5 is incorrect. The authors have provided a corrected version here.

**Fig 5:**
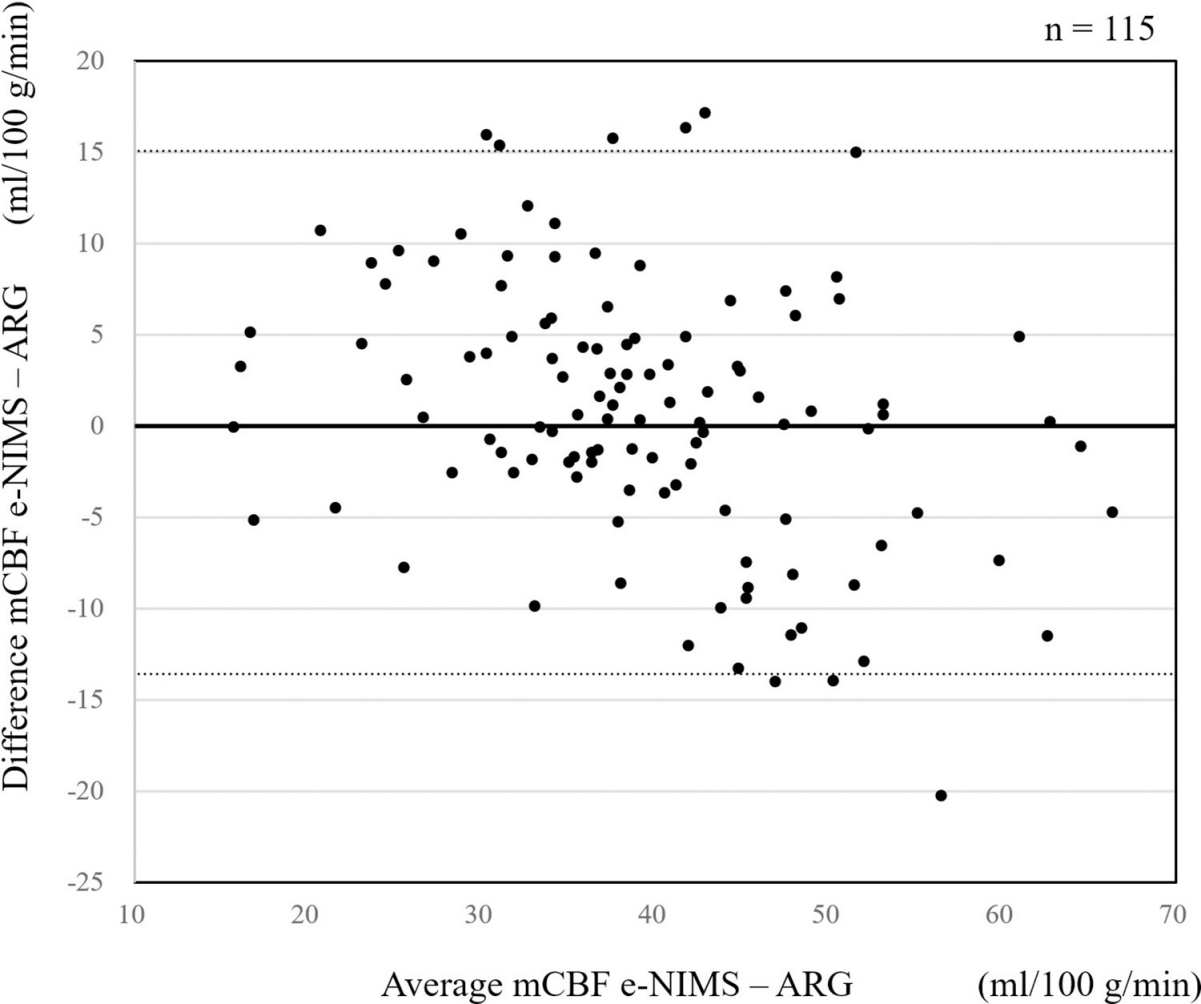
Bland-Altman plot for comparison of easy non-invasive micro sphere and autoradiography. Small dashed line denotes the limits of agreement (± 1.96 SD).
